# RNA Sequencing of Epithelial Cell/Fibroblastic Foci Sandwich in Idiopathic Pulmonary Fibrosis: New Insights on the Signaling Pathway

**DOI:** 10.3390/ijms23063323

**Published:** 2022-03-19

**Authors:** Fiorella Calabrese, Francesca Lunardi, Veronica Tauro, Federica Pezzuto, Francesco Fortarezza, Luca Vedovelli, Eleonora Faccioli, Elisabetta Balestro, Marco Schiavon, Giovanni Esposito, Stefania Edith Vuljan, Chiara Giraudo, Dario Gregori, Federico Rea, Paolo Spagnolo

**Affiliations:** 1 Department of Cardiac, Thoracic, Vascular Sciences and Public Health, University of Padova, 35128 Padova, Italy; francesca.lunardi@unipd.it (F.L.); veronicatauro@hotmail.it (V.T.); federica.pezzuto@unipd.it (F.P.); luca.vedovelli@unipd.it (L.V.); faccioli.eleonora@gmail.com (E.F.); marcoschi79@yahoo.it (M.S.); stefvu@hotmail.it (S.E.V.); dario.gregori@unipd.it (D.G.); federico.rea@unipd.it (F.R.); paolo.spagnolo@unipd.it (P.S.); 2 Padova University Hospital, 35128 Padova, Italy; francesco.fortarezza@unipd.it (F.F.); elisabetta_balestro@hotmail.com (E.B.); 3 Venetian Institute of Oncology IOV-IRCCS, 35128 Padova, Italy; giovanni.esposito@iov.veneto.it; 4 Department of Medicine, University of Padova, 35128 Padova, Italy; chiara.giraudo@unipd.it

**Keywords:** idiopathic pulmonary fibrosis, RNA-sequencing, transcriptomic, fibroblastic foci

## Abstract

Idiopathic pulmonary fibrosis (IPF) is a progressive and fatal lung disease characterized by irreversible scarring of the distal lung. IPF is best described by its histopathological pattern of usual interstitial pneumonia (UIP), characterized by spatial heterogeneity with alternating interstitial fibrosis and areas of normal lung, and temporal heterogeneity of fibrosis characterized by scattered fibroblastic foci (FF), dense acellular collagen and honeycomb changes. FF, comprising aggregated fibroblasts/myofibroblasts surrounded by metaplastic epithelial cells (EC), are the cardinal pathological lesion and their presence strongly correlates with disease progression and mortality. We hypothesized that the EC/FF sandwich from patients with UIP/IPF has a distinct molecular signature which could offer new insights into the crosstalk of these two crucial actors in the disease. Laser capture microdissection with RNAseq was used to investigate the transcriptome of the EC/FF sandwich from IPF patients versus controls (primary spontaneous pneumothorax). Differentially expressed gene analysis identified 23 up-regulated genes mainly related to epithelial dysfunction. Gene ontology analysis highlighted the activation of different pathways, mainly related to EC, immune response and programmed cell death. This study provides novel insights into the IPF pathogenetic pathways and suggests that targeting some of these up-regulated pathways (particularly those related to secreto-protein/mucin dysfunction) may be beneficial in IPF. Further studies in a larger number of lung samples, ideally from patients with early and advanced disease, are needed to validate these findings.

## 1. Introduction

Idiopathic pulmonary fibrosis (IPF) is a progressive and lethal lung disorder characterized by an aberrant remodeling of lung parenchyma following persistent alveolar epithelial injury [1]. Current guidelines on the treatment of IPF recommend the use of pirfenidone and nintedanib; however, they only slow rather than halt disease progression and do not significantly improve a patient’s quality of life. Indeed, the use of these drugs may incur high out-of-pocket costs without changing the overall progression of the disease and the high mortality within 3 to 5 years after diagnosis [2]. Thus, there is an urgent need to develop novel and more efficacious therapeutic strategies. Yet, this requires a better understanding of disease pathobiology. The etiopathogenesis of IPF is poorly understood; however, it is hypothesized that repeated injuries to alveolar epithelial cells (EC) are the key initiating factor that leads to excessive and sustained fibroblast activation and subsequent accumulation of matrix-producing myofibroblasts. The histopathological pattern of IPF is usual interstitial pneumonia (UIP), a morphologic entity defined by spatial heterogeneity with alternating patchy interstitial fibrosis and areas of normal lung, temporal heterogeneity of fibrosis characterized by scattered fibroblastic foci (FF) in the midst of dense acellular collagen and architectural alterations due to chronic scarring or honeycomb changes. FF, comprising aggregates of fibroblasts and myofibroblasts surrounded by metaplastic EC, represents the cardinal pathological lesion of active fibrogenesis [3]. Several studies have found a strong correlation between the presence/number of FF and both disease progression and mortality [4,5,6,7,8,9]. The sandwich of EC covering FF is the area of aberrant epithelial–mesenchymal crosstalk wherein an imbalance between profibrotic and antifibrotic mediators is thought to occur. Several high throughput technologies have been used to identify novel genes and pathways involved in human IPF lung tissue [10]. The recent development of RNA sequencing has enabled routine profiling of the whole transcriptome including coding and non-coding RNAs, detection of large dynamic ranges of transcripts, and identification of novel transcripts and variants [11,12]. To date, few studies have investigated IPF lung tissues by using this methodology, mostly without selecting a specific area of the lungs [13,14,15,16,17,18,19]. 

Laser capture microdissection (LCM) of IPF lung tissue represents an ideal approach to selectively isolate small tissue areas, even isolated cells. A recent study by Guillotin and colleagues, who performed transcriptome analysis and employed a new bioinformatic approach in FF isolated from lung tissues by LCM, identified prominent clusters of genes associated with cell cycle, inflammation/differentiation, translation, and cytoskeleton/cell adhesion. Additional studies focusing on collagen analysis identified some key signaling networks associated with fibrogenesis [20]. 

We hypothesized that the epithelial cell/fibroblastic foci (EC/FF) sandwich from patients with UIP/IPF has a distinct molecular signature that, if known, could provide new insights into the crosstalk of these two crucial actors in disease development, thus potentially defining novel therapeutic targets. This work has previously been presented in abstract form [21].

## 2. Materials and Methods

### 2.1. Study Population

This retrospective observational study included lung tissue from consecutive patients either undergoing lung transplantation for end-stage disease (*n* = 10) or video-assisted thoracoscopic surgical (VATS) biopsy for diagnostic purposes (*n* = 2) with a diagnosis of UIP/IPF obtained in 2019–2020. The diagnosis of UIP/IPF was reached after a multidisciplinary discussion in accordance with current international guidelines [22]. Microdissections of similar EC/FF sandwiches from patients who underwent surgical resection for primary spontaneous pneumothorax (*n* = 4) were used as controls. EC/FF sandwiches of both cases and controls were carefully selected according to the following criteria: (1) distant at least 200 µm from pleural surface, (2) EC/FF sandwich area of at least 3000 µm^2^, (3) presence of EC and fibroblasts confirmed by specific immunohistochemistry (cytokeratin—CK 7 for EC and vimentin for fibroblasts). 

The research was carried out in accordance with the principles of the Helsinki Declaration of 1975 as revised in 1983 and the guidelines for Good Clinical Practice. The Institutional ethics committee approved the study (4280/AO/17) and all patients gave their consent to the research.

### 2.2. LCM and Extraction of RNA

**Figure 1:**
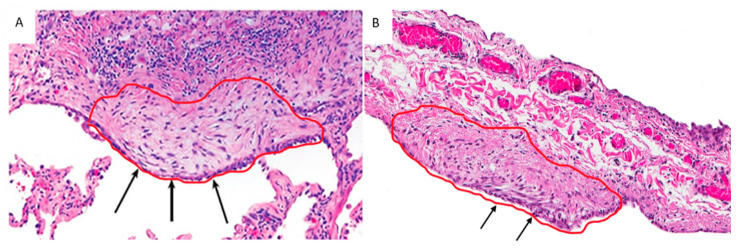
EC/FF sandwich area selected from a UIP/IPF patient (**A**) and a control (primary spontaneous pneumothorax) (**B**). Arrows show a similar structure, characterized by the presence of EC lining aggregates of fibroblasts (confirmed by immunohistochemistry).

Formalin-fixed paraffin-embedded (FFPE) blocks were cut into 4–8 μm thick sections and processed with standard hematoxylin-eosin staining. RNase contamination was avoided by cleaning surfaces and tools with RNaseZap™ RNase Decontamination Solution (ThermoFisher Scientific, Waltham, Massachusetts, USA). EC/FF sandwiches were identified by an experienced pathologist (FC) and captured by a Leica LMD6500 laser microscope (Leica Microsystems, Wetzlar, Germany) cutting into a 0.5 mL tube cap (Eppendorf, Hamburg, Germany) (Figure 1). Care was taken to avoid capturing other cell components (i.e., inflammatory cells). For UIP/IPF cases, mean (range) value of 8.3 (3–22) EC/FF sandwiches per section were captured and 8–15 sections/patient were used (the mean (±SD) total number of microdissected EC/FF sandwiches was 86.5(±31)). For controls, mean (range) value of 5.2 (3–9) EC/FF sandwiches per section were captured and 16 sections/patient were used (the mean (±SD) total number of microdissected EC/FF sandwiches was 81(±42)). Total RNA was extracted immediately after microdissection using RNeasy^®^ FFPE kit (Qiagen, Hilden, Germany) according to the manufacturer’s protocol. RNA yield and quality were determined by UV adsorption on a nanoDrop 1000 spectrophotometer and fragment size was analyzed using the RNA 6000 Pico assay (Agilent Technologies, Santa Clara, CA, USA) run on the 2100 Bioanalyzer. RNA quality was assessed using DV200 values and all cases had DV200 ≥ 39% and were included for library preparation.

### 2.3. Library Preparation and RNA Sequencing

SMARTer Stranded Total RNA-Seq Kit—Pico Input Mammalian (Takara Bio USA, Inc., Mountain View, CA, USA) was used to generate sequencing libraries following the manufacturer’s instructions. Twenty amplification cycles were required to obtain sufficient library concentration for sequencing. cDNA concentration was determined using the Qubit Fluorometer (Life Technologies, Carlsbad, CA, USA) and the quality was tested using the Agilent 2200 Tapestation system (Agilent Technologies, Santa Clara, CA USA). Cluster generation was achieved according to the Illumina Cluster Generation User and cDNA libraries were sequenced on a flow cell with a paired-end sequencing 2 × 150 bp with a data yield of 1300–1500 Gb on a HiSeq 4000 System sequencer (Illumina, San Diego, CA, USA).

### 2.4. RNA Sequencing Data Analyses

A quality check was performed on the raw sequencing data, removing low-quality portions while preserving the longest high-quality part of NGS reads. This trimming step was performed setting the minimum length to 35 bp and the quality score to 25 using the software BBDuk. High-quality reads were then aligned with the human reference genome sequence (GRCh38). After calculating gene expression values as raw fragment counts by FeatureCounts (version 1.5.1), a normalization was applied to the raw fragment counts by using the Trimmed Mean of M-values (TMM) and the Fragments Per Kilobase Million (FPKM) normalization. Cases and controls were compared using R with the package NOIseq and with the HTSFilter package to remove lowly expressed genes; similarly, among cases, we compared from video-assisted thoracoscopic surgery (VATS) and explanted lungs. The overall quality of the experiment was evaluated, based on the similarity between replicates, by a principal component analysis (PCA) using normalized gene expression values as input. We applied the Benjamin–Hochberg correction for false discovery rate (*p* < 0.05) and the statistically significant genes were used to generate an MA plot, a volcano plot, and a heatmap to show the expression profile of the differentially expressed genes. The most important ones were detected also by immunohistochemistry with monoclonal antibodies according to the manufacturer’s instructions. Gene ontology enrichment analysis (GOEA) was performed to identify the most enriched gene ontology (GO) categories across the differentially expressed genes (DEGs). GOEA was divided into three major functional categories (molecular function, cellular component, and biological process) and further analysis was carried out using PANTHER [23]. 

### 2.5. Immunohistochemistry

Immunohistochemistry (IHC) was performed for some of the overexpressed genes (MUC5B, MUC5AC, KRT5, KRT6A, KRT17, KRT19) following the antibody manufacturer’s protocol. Three μm thick sections were processed for immunohistochemical analysis using MUC5B monoclonal antibody (clone 4A10-H2-Novus Biologicals, Centennial, CO, USA), MUC5AC (clone CLH2, Leica Biosystems, Milan, Italy), KRT5 (clone XM26, Leica Biosystems, Milan, Italy), KRT6A (clone KRT6A/2368, Abcam, Cambridge, UK), KRT17 (clone E3, Leica Biosystems, Milan, Italy), KRT19 (clone B170, Leica Biosystems, Milan, Italy). IHC was performed in the Leica Bond-III Autostainer (Leica Microsystems Srl, Wetzlar, Germany) according to the manufacturer’s protocol. Finally, the sections were counterstained with Mayer‘s hematoxylin.

## 3. Results

### 3.1. Study Population, LCM, and RNA Extraction

The main clinical and morphological characteristics of the study population are reported in Table 1. All RNA samples had an optimal ratio of quality/concentration to be used in the RNA-Seq library preparation, without any significant difference between cases and controls. 

Consistent with previous reports, the integrity of RNA isolated from FFPE tissues is decreased probably due to formalin fixation and archival time. All RNA isolations had OD260/280 >1.9 confirming the high purity of RNA. The RIN numbers were in the range of 2.1–2.6 and the most abundant RNA fragments were in the range of 100–150 ribonucleotides for all samples, suggesting a similar degradation and quality regardless of whether FFPE tissues were obtained from controls or UIP/IPF patients. Considering all microdissected cases, the mean (±SD) DV200 values were 54 (±11): in particular, 56% of the samples showed a medium-high quality (values ranging from 52% to 72%). Concerning quantity, mean (±SD) RNA concentration was 2353 (±2409) pg/μL: 2889 (±1765) pg/μL for UIP/IPF cases and 746 (±534) pg/μL for controls (Table 1).

**Figure 2:**
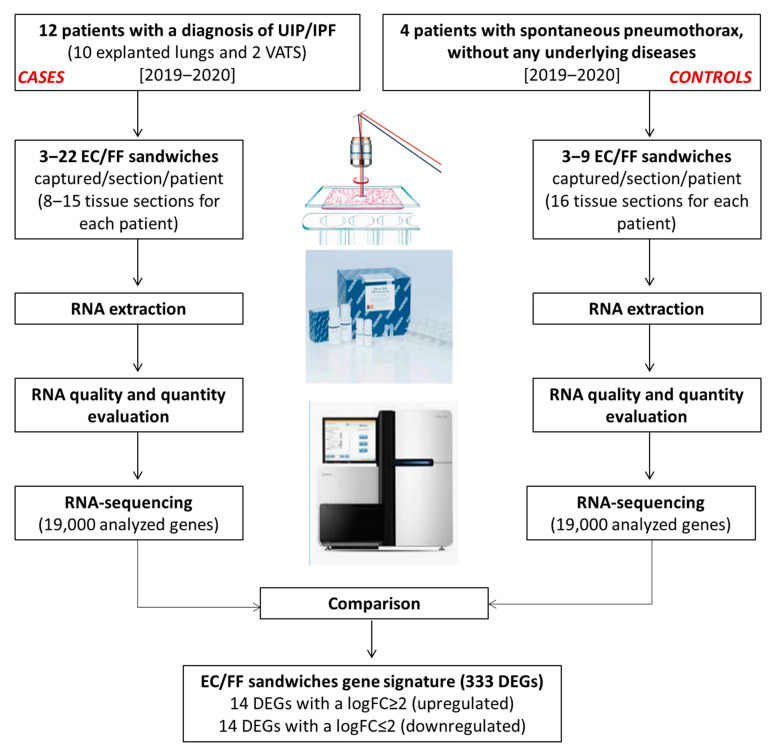
Generation of the EC/FF sandwich gene signature.

The study design and generation process of the EC/FF sandwich gene signature is shown in Figure 2. 

### 3.2. Comprehensive Profiling of UIP/IPF Cases Reveals Specific Alterations

**Figure 3:**
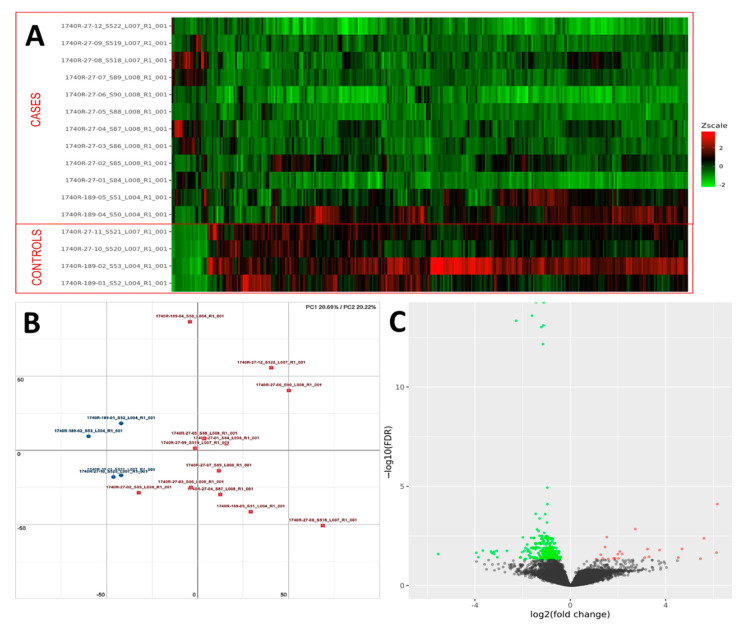
(**A**) Heatmap representation of the expression profile of the differentially expressed genes. A green-red gradient was used to show the scaled TMM values across the filtered samples. (**B**) PCA conducted on the normalized gene expression values of samples. *X*- and *Y*-axes represent two components, PC1 and PC2, respectively, with the amount of variance explained by each component being reported. Each point in the plot represents a sample: red squares indicate cases and blue circles controls. (**C**) A volcano plot shows the relationship between the fold-change (on the *X*-axis) and the significance of the differential expression test (*Y*-axis) for each gene in the genome. The distribution of the dots in the volcano plot can be useful to check whether a range of fold-changes is associated with a stronger or a weaker significance of differential expression. Black dots represent the genes that are not significantly differentially expressed, while red and green dots represent the genes that are significantly up- and down-regulated, respectively.

**Figure 4:**
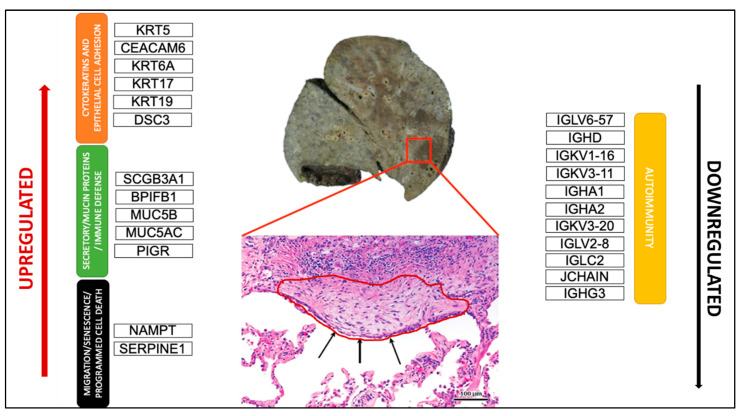
Schematic representation of the most crucial DEGs. Macroscopic view of the explanted lung of a UIP/IPF patient (transplant n°380). Arrows indicate metaplastic EC lining FF in the EC/FF sandwich (red circle).

PCA analysis of the RNAseq data was performed to comparatively characterize the overall transcriptome profiles. To this end, the PCA plot reveals distinct expression profiles corresponding to controls and cases, although the explained variance was low (21%) (Figure 3). Differential expression analysis showed that only 333 out of more than 19,000 genes analyzed had a significantly different expression (DEGs) in UIP/IPF cases vs. controls: 23 of them were up-regulated and 310 down-regulated (Spreadsheet S1). We focused on DEGs with a logFC ≥2 (if up-regulated) and ≤2 (if down-regulated) and the genes are listed in Table 2 and Table 3. The overall distribution of these transcriptome changes in relation to the group was further characterized by hierarchical clustering and shown by a heatmap (Figure 3). In particular, the most up-regulated genes were SCGB3A1, BPIFB1, MUC5B, MUC5AC, KRT5, KRT6A, PIGR, MMP13, KRT17, DSC3, CEACAM6, KRT19, NAMPT, and SERPINE1. The most down-regulated genes were those coding immunoglobulin chains (e.g., IGLV6-57, IGHD, IGKV1-16), SLC40A1, DEPTOR, and SMOC2 (Figure 4). 

**Figure 5:**
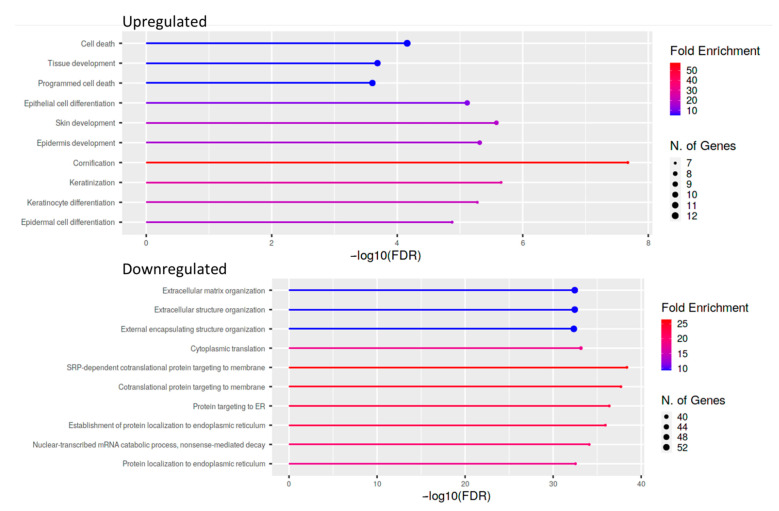
Lollipop plot of the ten most enriched pathways for GO Biological process with FDR <0.05. Significant DEGs were related to known biological, molecular, and cellular processes including cell death and epithelial cell differentiation, based on GO enrichment profile.

**Figure 6:**
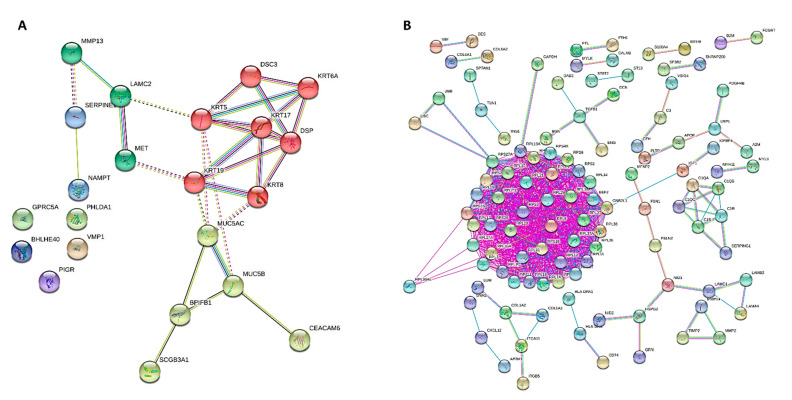
PPI network of DEGs for GOBP obtained from STRING, showing how the genes are inter-connected ((**A)**, up-regulated; (**B**), down-regulated). Up-regulated and down-regulated gene enrichment data are in Tables S1 and S2.

For cases and controls, enrichment and network analysis of the 333 DEGs divided for up- and down-regulated were performed using ShinyGO v0.741 (http://bioinformatics.sdstate.edu/go/, accessed date: 13 December 2021) (Tables S1 and S2). Biological processes related to epithelial cell dysfunction with activation of innate immune response and programmed cell death were up-regulated in UIP/IPF cases (Figure 5). Conversely, down-regulated genes were related to transcription processes and molecular functions, such as extra-cellular matrix (ECM) constituents and factors involved in collagen and GAG binding. Network visualization of the enriched pathways derived from gene ontology obtained is shown in Figure 6 and Figure S1.

### 3.3. Immunohistochemistry

**Figure 7:**
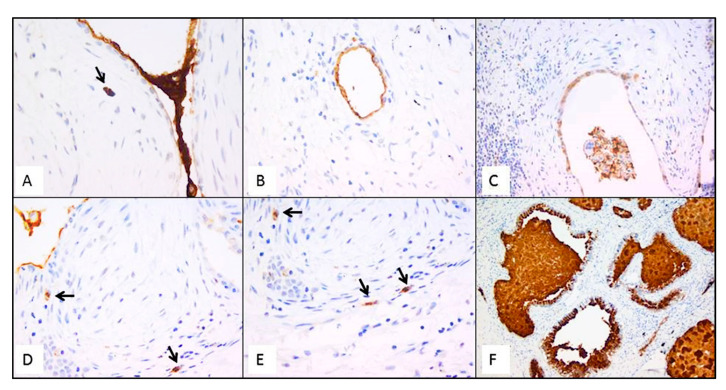
Representative futures of immunohistochemistry for MUC5B (clone 4A10-H2; 1:200, Novus Biologicals, Centennial, CO, USA) in emblematic UIP/IPF cases. Staining is visible in mucus between two EC/FF sandwiches and in the metaplastic EC lining FF (**A**); hyperplastic type II pneumocytes lining alveolar spaces (**B**); hyperplastic type II pneumocytes and macrophages (**C**); hybrid cells in FF areas (**D**,**E**); honeycomb cysts filled with mucus (**F**). Arrows indicate the hybrid cells in figure (**A**,**D**,**E**).

**Figure 8:**
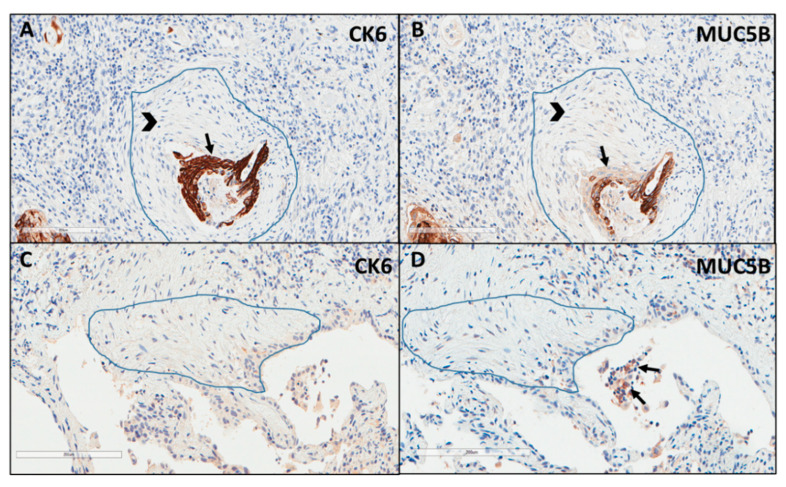
Explicative images of immunohistochemistry for KRT6 (CK6) (**A**) and MUC5B (**B**) in a UIP/IPF case and in a control patient (**C**,**D**). Strong immunostaining is well visible in the metaplastic EC (arrow) surrounding the FF (head arrow) (**A**,**B**) while completely negative in (**C**,**D**). Only some inflammatory cells (macrophages) of the alveoli show a weak MUC5B staining (arrows) (**D**).

Immunostaining of all mucins was strongly positive in UIP/IPF cases (Figure 7). MUC5B and MUC5AC positive immunostaining was mainly detected in metaplastic EC lining the distal bronchial tract, honeycomb lesions, and some hyperplastic type II pneumocytes lining alveolar spaces. In particular, the EC/FF sandwich, the area of our interest, showed strong immunostaining of metaplastic EC lining FF and some hybrid cells (cells with transitional features of both EC and fibroblasts) that were detected inside the FF areas of UIP/IPF patients (Figure 7). All investigated cytokeratins were strongly expressed in the UIP/IPF EC/FF sandwiches. EC/FF sandwiches in the controls were consistently negative for mucins and weak positivity was observed for cytokeratins. KRT6 (CK6) was always negative (Figure 8). 

### 3.4. Comprehensive Profiling of Early and Late UIP/IPF Stages Reveals Specific Alterations

**Figure 9:**
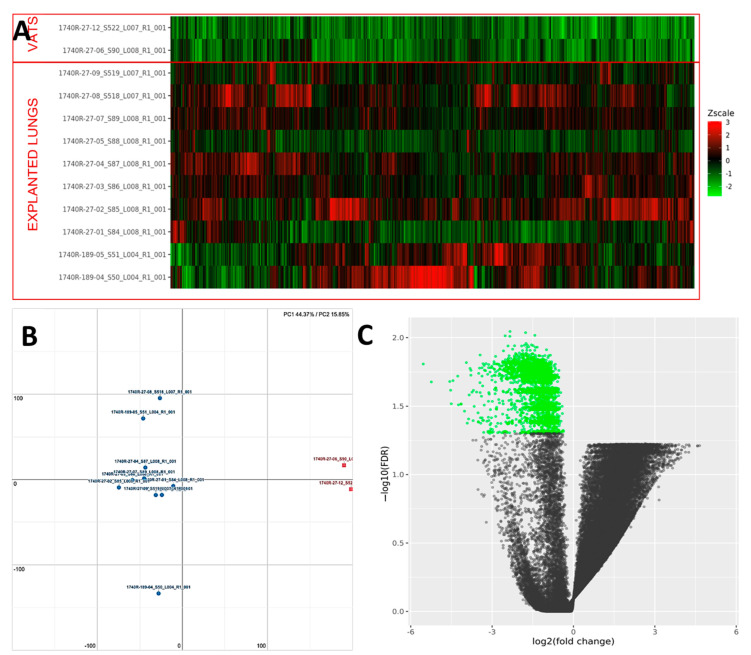
(**A**) Heatmap representation of the expression profile of the differentially expressed genes. A green-red gradient was used to show the scaled TMM values across the filtered samples. (**B**) Principal component analysis (PCA) conducted on the normalized gene expression values of samples. *X*- and *Y*-axes represent two components, PC1 and PC2, respectively, with the amount of variance explained by each component being reported. Each point in the plot represents a sample: red squares indicate VATS and blue circles explanted lungs. (**C**) A volcano plot shows the relationship between the fold-change (on the *X*-axis) and the significance of the differential expression test (*Y*-axis) for each gene in the genome. The distribution of the dots in the volcano plot can be useful to check whether a range of fold-changes is associated with a stronger or a weaker significance of differential expression. Black dots represent the genes that are not significantly differentially expressed, while green dots represent the genes that are significantly down-regulated.

PCA analysis of the RNAseq data reveals distinct expression profiles corresponding to early and late UIP/IPF cases. The explained variance along the PC1 axis was 44% and 16% along the PC2 (Figure 9). More than 2000 genes were found to be down-regulated in UIP/IPF cases with the early disease compared to cases with end-stage disease (Figure 9). The most down-regulated genes were ATP5IF1 and MUC5AC, with a LogFC <−5. 

## 4. Discussion

Our study found that transcriptomic analysis of RNA isolated from the FFPE EC/FF sandwich is feasible and may allow for identifying key transcriptional pathways. The LCM-based approach was optimized to select FF and EC conjointly (aggregates of fibroblast/myofibroblasts and covering metaplastic/transitional EC); indeed, investigating the sandwich as a single functional unit is more likely to discover key factors that modulate the epithelial/mesenchymal crosstalk. To this end, we micro-dissected this area using FFPE sections of 12 UIP/IPF patients. The mean (±SD) total was 86.5 ± 31 of the EC/FF sandwiches captured in each case identified a set of 333 DEGs (23 of which were up-regulated and 310 down-regulated). 

Previous studies have applied transcriptomics to IPF lung samples but used different approaches, thus identifying often a limited number of genes [24,25,26,27,28,29,30,31]. Moreover, transcriptomic analysis was mainly performed using RNA obtained from whole lung tissue, thus without separation of specific area/cell components. During the past decade, microarrays have represented the most widely used approach for transcriptome analysis, but high-throughput sequencing of cDNA (RNA-seq) has recently emerged as a powerful alternative. RNA-seq produces millions of short reads that are mapped to a reference genome. This method of DEGs analysis offers several advantages over microarrays, including the possibility to sequence the entire genome. Conversely, microarrays are based on specific probes that may detect only a limited number of sequences and require a large number of samples to obtain meaningful results. Previous methodological studies, including one from our group, have confirmed the feasibility of RNA-Seq in FFPE lung samples of IPF cases with high concordance between RNA-Seq on FFPE and microarray/RNAseq on snap-frozen fresh tissues [14,21]. To the best of our knowledge, this is the first transcriptome study carried out on highly selected lung tissue areas of IPF patients using an RNA-sequencing approach. 

DEGs analysis showed that only 333 out of more than 19,000 genes analyzed were significantly differentially expressed; 23 of them were up-regulated and 310 down-regulated. 

Guillotin et al. performed a similar study, yet with some major differences in the study design and methodology [20]. Specifically, they used LCM to select only FF, not the EC/FF sandwich, thus the differences we found could be due to the presence of the metaplastic EC surrounding the FF. In addition, they performed the transcriptome analysis by microarray, not RNA-sequencing; finally, the generation of FF gene signature was based on an informatics pooling strategy, not on the comparison with a control population as was instead the case in our study. The authors identified prominent clusters of genes associated with the cell cycle, inflammation/differentiation, translation, and cytoskeleton/cell adhesion. 

Our DEGs analysis identified 23 up-regulated genes and with more than half (14 out 23) showing a logFC >2 and mainly related to epithelial dysfunction.

The up-regulation of gene transcripts of several cytokeratins, such as KRT5, 6A, 17, and 19, as well as DSC3 (desmocollin-3: a glycoprotein member of the desmosomal family), highlights the importance of various transitional/intermediate and metaplastic epithelial cell phenotypes in EC/FF. Several studies have suggested a role for these cytokeratins in the pathobiology of IPF, with overexpressed KRT5, 6A, and 19 that were reported correlated with worsening disease severity and survival [32,33,34,35,36]. Following induction by tissue injury, these cytokeratins play a crucial role in epithelial–mesenchymal transition (EMT) and ECM production. A few recent studies using single-cell RNAseq found that the KRT17 gene is overexpressed in IPF, providing further support to the hypothesis that these cells are actively involved in the production of ECM components, including collagen [16,37,38]. Interestingly, the study by Adams TS et al. reported strong immunostaining of KRT17 in the metaplastic EC covering FF, as we consistently found in our cases [37].

Interestingly, in the EC/FF area, we found several up-regulated secretory/mucin components, which are generally produced by normal airway EC and exert anti-inflammatory activities, namely SCGB3A1, BPIFB1, MUC5B, and MUC5AC. Interestingly, many of these transcripts, especially mucins, were previously studied in IPF, although their exact role in the activation of profibrotic intracellular signaling remains to be elucidated. Mucins are high-molecular-weight glycoproteins produced by surface epithelium goblet cells, submucosal glands, and serous cells. They are classified into two groups depending on whether they are secreted (secreted mucins) or tethered to epithelial cell membranes (transmembrane mucins). Secreted mucins are further subdivided into gel-forming and non-gel-forming mucins. Mucins are encoded by MUC genes. To date, 21 human MUC genes have been identified of which 16 have been identified in the lung: 10 transmembrane mucins (MUC1, MUC4, MUC12, MUC13, MUC14, MUC15, MUC16, MUC20, MUC21, MUC22); 4 secreted gel-forming mucins (MUC2, MUC5AC, MUC5B, MUC19); and 2 secreted, non-gel-forming mucins (MUC7, MUC8) [39,40,41,42,43]. Together, MUC5AC and MUC5B account for approximately 90% of the airway mucus and dominate its biophysical properties. In addition, promoter polymorphisms within MUC5B (rs35705950) are the most important genetic risk factor for both familial and sporadic IPF, being present in about 40% of patients and 10% of controls, with both a predictive and prognostic role in lung fibrosis [44,45,46,47].

This polymorphism leads to an increased expression of MUC5B, and IPF patients, irrespective of their genetic background, show increased levels of MUC5B in the distal airways and in honeycomb cysts [48,49,50,51]. 

The mucin (MUC5AC, MUC5B) expression tissue analysis performed in our study by immunohistochemistry confirmed the extensive positive immunostaining, not only in distal airways and honeycomb cysts, as already described, but also in the transitional/metaplastic EC of sandwiches that represent the area of our major interest. Whether and to what extent MUC5B accumulation influences the development of lung fibrosis remains to be determined. However, a number of pathogenetic hypotheses related to impaired mucociliary clearance with local inflammation or abnormal epithelialization were proposed. Specifically, dysregulation of secretory components, particularly mucins, may result in chronic retention of several inhaled substances (air pollutants, cigarette smoke, microorganisms) and endogenous debris that lead to microscopic scaring and progressive lung fibrosis in temporally and spatially distinct areas. Alternatively, mucus overproduction could hamper alveolar repair, by interfering either with the interaction between EC and the underlying matrix or with the surface-tension properties of surfactant [50].

Aberrant expression of mucins was also associated with proliferation, altered cellular adhesion, and EMT. In IPF, determining the role of mucin-mediated EMT pathways in the development/progression of the disease requires further research. In vivo, experimental models have shown that several mucins (MUC1, MUC5b, MUC4) can influence EMT through different mechanisms, such as promoting fibroblast proliferation and migration while exerting also anti-apoptotic activities [52,53,54,55]. Indeed, their genetic and pharmacologic modulation was found to protect bleomycin-treated mice by interfering with TGF-β1-induced EMT or myofibroblast differentiation [56].

Mucin-mediated EMT is very complex and is better studied in several oncological diseases [55]. A cluster of carcinoembryonic antigen cell adhesion molecules (CEACAM5, CEACAM6, and CEACAM7) were found to be critical modulators of progression and invasion of several tumors [57,58]. CEACAM6, overexpressed in several cancers and in gastrointestinal tumors or in mucin-producing carcinoma [59], is implicated in metastatic activity by promoting EMT [60]. Although the mechanisms by which CEACAM6 regulates EMT are poorly understood, it was shown that CEACAM6 acts as an oncogene by promoting EMT in different tumor types. In our transcriptomic study, CEACAM6 was up-regulated in the IPF sandwich compared to controls. An interesting clinical study performed in patients with severe asthma showed overexpression of CEACAM6 in hyperplastic/metaplastic EC strictly correlated with MUC5B, thus suggesting an up-regulation of this glycoprotein in injured metaplastic cells [61].

Two additional important transcripts found differently expressed in patients and controls were NAMPT (Nicotinamide Phosphoribosyltransferase) and SERPINE 1 (also named Plasminogen activator inhibitor 1 -PAI-1). Both genes have several functions and are believed to play a key role in pulmonary fibrosis by regulating cell adhesion, migration, senescence, and apoptosis. Several experimental models have found that lung fibrosis may be significantly modified by modulating these genes; in particular, reductions in NAMPT expression facilitate IPF myofibroblast apoptosis and lead to protection from fibrosis in vivo [62], whereas overexpression of PAI-1 enhances lung fibrotic responses [63,64,65,66,67,68,69,70]. Both studies concluded that the removal of senescent cells ameliorates fibrosis in animal models and is considered to be a potential therapeutic approach in lung fibrosis.

Among up-regulated gene transcripts in this area, the only one more strictly related to ECM turnover was MMP13 (matrix metalloproteases). The contribution of MMPs to the pathogenesis of IPF is well known but their specific roles remain unclear. Increased levels of several MMPs (MMP1, MMP7, MMP8, and MMP9) were found in the blood, bronchoalveolar lavage fluid, and lung samples from patients with IPF [71]. MMP13 is a member of the collagenase family, which degrades type I, II, III, X, and XIV collagens, tenascin C, fibronectin, aggrecan, osteonectin, perlecan, versican, and fibrillin 1. While contradictory data about the role of MMP13 in promoting or attenuating fibrosis were reported in experimental models, clinical data showed elevated mRNA and protein levels in IPF lung tissue in several parts of the alveolar and bronchiolar epithelium, alveolar septa, and interstitial spaces [72]. However, it is also possible that up-regulated MMP13 in the EC/FF sandwich exerts effects beyond its catalytic activity in substrates that are not components of ECM. 

Our study has several strengths. Performing high-throughput analysis requires an appropriate selection of cases and a careful analysis of the biological materials to be processed, similar to what is currently performed in oncology. For this reason, our study included highly selected active areas of the disease from well-characterized UIP/IPF patients diagnosed in a multidisciplinary team based on the most recent guidelines [22]. 

Moreover, we used FFPE lung sections to make sure we selected precisely the EC/FF sandwiches. This process would not have been so precise if we had used frozen sections. Another important strength is the availability of EC/FF control sandwiches. Although these samples had some limitations (e.g., samples coming from younger patients), they were likely as close to the IPF sandwich as could be pragmatically possible. Some limitations should also be acknowledged. First, we acknowledge that our study includes a relatively small number of IPF patients and controls, however, it is the first study that performed LCM-RNA seq and was able to identify a discriminatory transcriptomic signature from this critical area of the IPF lung. However, even in this relatively small cohort, we were able to identify a discriminatory transcriptomic signature from this critical area of the IPF lung. Second, the majority of our cases included samples from patients with advanced diseases who underwent lung transplantation. We observed some differences between late and early UIP/IPF but we need a larger number of cases, mainly early, to confirm these interesting data. In particular, the inclusion of a larger number of patients would also help to investigate the influence of treatment in gene expression. Moreover, it could be useful to include as a control group not only not fibrotic patients as those affected by primary spontaneous pneumothorax but also patients suffering from other types of fibrotic interstitial lung diseases, such as connective tissue disease and/or chronic hypersensitive pneumonitis.

## 5. Conclusions

In summary, the present study provides strong evidence for the use of high throughput technologies in crucial areas of UIP/IPF lungs. We identified up-regulation of several transcriptional pathways mainly related to epithelial dysfunction. The GO analysis highlighted the activation of different pathways, mainly related to EC, immune response, and programmed cell death, suggesting that targeting some of these up-regulated pathways (particularly those related to secreto-proteins/mucin dysfunction) may represent a potential therapeutic option for IPF. These results merit further examination using a larger number of lung samples coming from both early and advanced diseases. We encourage researchers involved in this field to include more archival tissues in their studies, especially selecting specific areas, because these samples are easily obtainable and represent a valuable resource for RNA expression signatures.

## Figures and Tables

**Table 1:** Main demographic and clinical data of the study population.

	Study Population (16 Patients)	UIP/IPF Cases (12 Patients)	Controls (4 Patients)
Age (years) [mean ± SD]	51 (±16.66)	58.33 (±7.82)	29 (±16.77)
Sex (males) [N (%)]	16 (100%)	12 (100%)	4 (100%)
Type of specimen			
Explanted lung	10 (62.5%)	10 (83%)	0 (0%)
Diagnostic surgical biopsy	6 (37.5%)	2 (17%)	4 (100%)
Number of microdissected EC/FF sandwiches [mean ± SD]	84 ±37	86.5 ± 31	81 ± 42
RNA quantity (pg/μL) [mean ± SD]	2353 ± 2409	2889 ± 1765	746 ± 534
RNA quality (DV200, %) [mean ± SD]	54 ± 11	50 ± 25	66 ± 5

**Table 2:** List of up-regulated genes with a logFC ≥ 2.

Gene Name	Gene ID	logFC	FDR	Gene Description
SCGB3A1	ENSG00000161055	6.17	7.8 × 10^−5^	Secretoglobin family 3A member 1 [Uniprot/SWISSPROT Acc. Q96QR1]
BPIFB1	ENSG00000125999	6.13	0.02	BPI1 domain-containing protein [Uniprot/SPTREMBL Acc. A2A2R0]; BPI fold-containing family B member 1 [Uniprot/SWISSPROT Acc. Q8TDL5]
MUC5B	ENSG00000117983	5.6	0.004	Mucin-5B [Uniprot/SWISSPROT Acc. Q9HC84]
MUC5AC	ENSG00000215182	5.5	0.05	Mucin-5AC [Uniprot/SWISSPROT Acc. P98088]
KRT5	ENSG00000186081	4.7	0.01	IF rod domain-containing protein [Uniprot/SPTREMBL Acc. F8W0C6]; IF rod domain-containing protein [Uniprot/SPTREMBL Acc. H0YI76]; IF rod domain-containing protein [Uniprot/SPTREMBL Acc. H0YIN9]; Keratin, type II cytoskeletal 5 [Uniprot/SWISSPROT Acc. P13647]
KRT6A	ENSG00000205420	4.5	0.04	IF rod domain-containing protein [Uniprot/SPTREMBL Acc. A0A0S2Z428]; Keratin, type II cytoskeletal 6A [Uniprot/SWISSPROT Acc. P02538]
PIGR	ENSG00000162896	3.8	0.02	Polymeric immunoglobulin receptor [Uniprot/SWISSPROT Acc. P01833]
MMP13	ENSG00000137745	3.3	0.04	Collagenase 3 [Uniprot/SPTREMBL Acc. G5E971]; Collagenase 3 [Uniprot/SWISSPROT Acc. P45452]; Collagenase 3 [Uniprot/SPTREMBL Acc. Q7Z5M0]
KRT17	ENSG00000128422	3.2	0.015	IF rod domain-containing protein [Uniprot/SPTREMBL Acc. A0A3B3IS58]; IF rod domain-containing protein [Uniprot/SPTREMBL Acc. F5GWP8]; IF rod domain-containing protein [Uniprot/SPTREMBL Acc. K7EPJ9]; IF rod domain-containing protein [Uniprot/SPTREMBL Acc. K7ESE1]; Keratin, type I cytoskeletal 17 [Uniprot/SWISSPROT Acc. Q04695]
DSC3	ENSG00000134762	3.1	0.04	Desmocollin-3 [Uniprot/SPTREMBL Acc. J3QRL9]; Desmocollin-3 [Uniprot/SWISSPROT Acc. Q14574]
CEACAM6	ENSG00000086548	2.7	0.001	Carcinoembryonic antigen-related cell adhesion molecule 6 [Uniprot/SWISSPROT Acc. P40199]
KRT19	ENSG00000171345	2.2	0.03	IF rod domain-containing protein [Uniprot/SPTREMBL Acc. C9JM50]; IF rod domain-containing protein [Uniprot/SPTREMBL Acc. K7EMS3]; Keratin, type I cytoskeletal 19 [Uniprot/SWISSPROT Acc. P08727]
NAMPT	ENSG00000105835	2.1	0.02	Nicotinamide phosphoribosyltransferase [Uniprot/SPTREMBL Acc. A0A024R718]; Nicotinamide phosphoribosyltransferase [Uniprot/SPTREMBL Acc. A0A0C4DFS8]; Nicotinamide phosphoribosyltransferase [Uniprot/SWISSPROT Acc. P43490]
SERPINE1	ENSG00000106366	2.0	0.04	SERPIN domain-containing protein [Uniprot/SPTREMBL Acc. A0A024QYT5]; Plasminogen activator inhibitor 1 [Uniprot/SWISSPROT Acc. P05121]

**Table 3:** List of down-regulated genes with a logFC ≤ 2.

Gene Name	Gene ID	logFC	FDR	Gene Description
IGLV6-57	ENSG00000211640	−5.5	0.03	Immunoglobulin lambda variable 6–57 [Uniprot/SWISSPROT Acc. P01721]
IGHD	ENSG00000211898	−3.9	0.02	Immunoglobulin heavy constant delta [Uniprot/SPTREMBL Acc. A0A0A0MS09]; Immunoglobulin heavy constant delta [Uniprot/SWISSPROT Acc. P01880]
IGKV1-16	ENSG00000240864	−3.9	0.04	Immunoglobulin kappa variable 1–16 [Uniprot/SWISSPROT Acc. P04430]
IGKV3-11	ENSG00000241351	−3.7	0.02	Immunoglobulin kappa variable 3–11 [Uniprot/SWISSPROT Acc. P04433]
IGHA1	ENSG00000211895	−3.2	0.02	Immunoglobulin heavy constant alpha 1 [Uniprot/SWISSPROT Acc. P01876]
IGHA2	ENSG00000211890	−3.2	0.03	Immunoglobulin heavy constant alpha 2 [Uniprot/SPTREMBL Acc. A0A286YEY5]; Immunoglobulin heavy constant alpha 2 [Uniprot/SWISSPROT Acc. P01877]
IGKV3-20	ENSG00000239951	−3.1	0.02	Immunoglobulin kappa variable 3–20 [Uniprot/SWISSPROT Acc. P01619]
IGLV2-8	ENSG00000278196	−3.1	0.04	Immunoglobulin lambda variable 2–8 [Uniprot/SWISSPROT Acc. P01709]
IGLC2	ENSG00000211677	−2.7	0.02	Immunoglobulin lambda constant 2 [Uniprot/SWISSPROT Acc. P0DOY2]
SLC40A1	ENSG00000138449	−2.3	4.5–14	Solute carrier family 40 protein [Uniprot/SPTREMBL Acc. E7EQF8]; Solute carrier family 40 protein [Uniprot/SPTREMBL Acc. E7ES28]; Solute carrier family 40 member 1 [Uniprot/SWISSPROT Acc. Q9NP59]
JCHAIN	ENSG00000132465	−2.1	0.04	Immunoglobulin J chain [Uniprot/SWISSPROT Acc. P01591]
IGHG3	ENSG00000211897	−2.1	0.04	Immunoglobulin heavy constant gamma 3 [Uniprot/SPTREMBL Acc. A0A286YES1]
DEPTOR	ENSG00000155792	−2.0	0.02	DEP domain-containing mTOR-interacting protein [Uniprot/SWISSPROT Acc. Q8TB45]
SMOC2	ENSG00000112562	−2.0	0.004	SPARC-related modular calcium-binding protein 2 [Uniprot/SPTREMBL Acc. A0A087WTM0]; EF-hand domain-containing protein [Uniprot/SPTREMBL Acc. H0Y3J4]; EF-hand domain-containing protein [Uniprot/SPTREMBL Acc. H0Y5I1]; SPARC-related modular calcium-binding protein 2 [Uniprot/SWISSPROT Acc. Q9H3U7]

## Data Availability

Raw data are available in SRA.

## Supplementary Materials

The following supporting information can be downloaded at: https://www.mdpi.com/article/10.3390/ijms23063323/s1.
