# Supplementary figures and images for: RNA Sequencing of Epithelial Cell/Fibroblastic Foci Sandwich in Idiopathic Pulmonary Fibrosis: New Insights on the Signaling Pathway
Source: Int J Mol Sci. 2022 Mar 19;23(6):3323. doi: 10.3390/ijms23063323 (PMC8954546; doi:10.3390/ijms23063323)

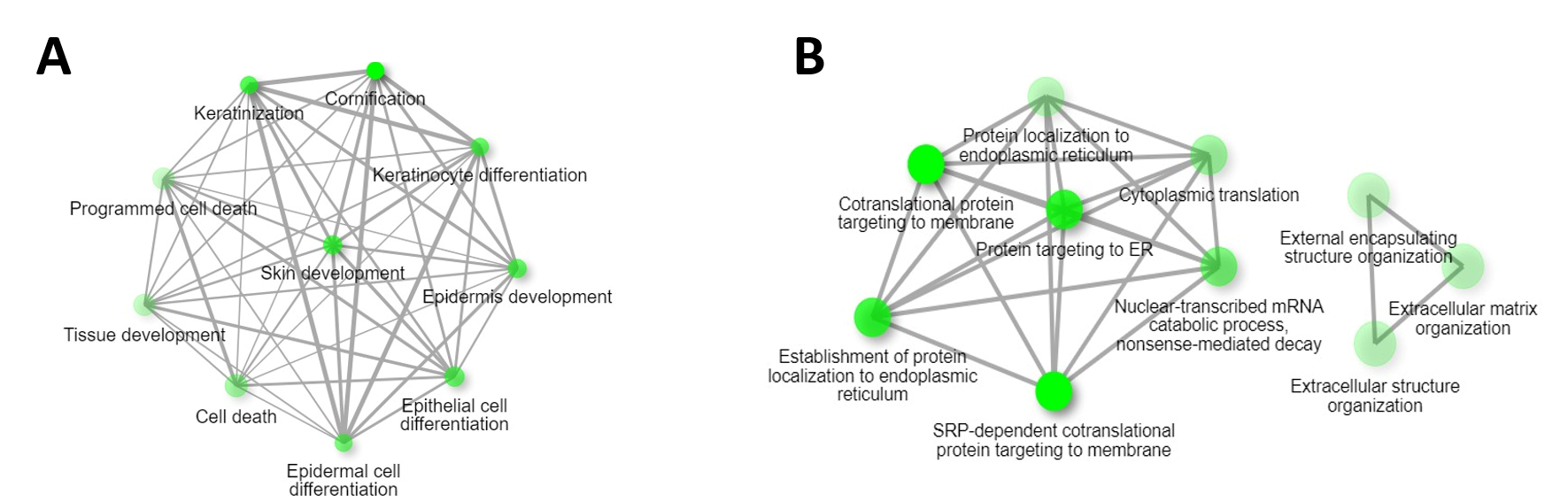

Supplement: Supplementary file 1 [file ijms-23-03323-s001.zip › Figure S1.tif]
